# Promising perspectives towards regrowing a human arm

**DOI:** 10.1007/s10856-013-5048-5

**Published:** 2013-09-29

**Authors:** Malgorzata Lewandowska-Szumiel, Ilona Kalaszczynska

**Affiliations:** Tissue Engineering Lab, Department of Histology and Embryology, Center of Biostructure Research, Medical University of Warsaw, Chalubinskiego 5, 02-004 Warsaw, Poland

## Abstract

Despite the great enthusiasm about tissue engineering during the 1980s and the many significant basic observations made since then, the clinical application of tissue-engineered products has been limited. However, the prospect of creating new human tissues and organs is still exciting and continues to be a significant challenge for scientists and clinicians. A human arm is an extremely complicated biological construction. Considering regrowing a human arm requires asking about the current state-of-the-art of tissue engineering and the real capabilities that it may offer within a realistic time horizon. This work briefly addresses the state-of-the-art in the fields of cells and scaffolds that have high regenerative potential. Additional tools that are required to reconstruct more complex parts of the body, such as a human arm, seem achievable with the already available more sophisticated culture systems including three-dimensional organization, dynamic conditions and co-cultures. Finally, we present results on cell differentiation and cell and tissue maturation in culture when cells are exposed to mechanical forces. We postulate that in the foreseeable future even such complicated structures such as a human arm will be regrown in full in vitro under the conditions of a mechanically controlled co-culture system.

## Introduction

Responding to Joachim Kohn’s challenge to consider the ability of regrowing a human arm, we naturally turned to regenerative medicine. According to the most concise description given by Mason, “regenerative medicine replaces or regenerates human cells, tissue or organs to restore or establish normal function” [1]. The concept we propose is based on the possibility of obtaining ex vivo a functional construction, which mimics both the appearance and function of a human limb. It would consist of a temporary scaffold and the patient’s own biological material. Our optimism is based on the opportunities offered by modern tissue engineering (TE). Already 15 years ago, McCarthy cited Robert Langer: “…Tissue engineering is at the stage that genetic engineering was at in 1981—no products approved, but some on the horizon. It is a potentially explosive area…” [2]. To the disappointment of many of us, the current number of clinical applications of TE products is still modest.

The majority of TE products have been introduced on the medical market in the United States of America (USA) [3]. In the European Union (EU), the appropriate approval procedure was regulated only at the end of 2008 [4]. This new policy may open new possibilities, although TE products have been classified in the EU as medicinal products and as such are subject to a costly and time consuming approval procedure. At the same time, significant scientific progress is being made in all fields that contribute to regenerative medicine: stem cell research, sophisticated scaffolds for cell manipulation and transplantation, and complex co-culture systems in vitro. In this paper, we present a brief review of the key areas of progress that bring us closer to successfully regrowing a human arm.

## From replacement to regeneration

The question—can we or can we not regrow a human arm?—came at a moment when, after years of investigations, biomaterials have been successfully implemented in the clinical setting as replacements for various tissues.

During the last two decades, biomaterial scaffolds especially designed for TE applications have been proposed [5]. Interestingly, although a huge variety of synthetic implantable materials is available, the majority of TE products on the medical market are based on the scaffolds of natural origin (Table 1). This indicates that the real expansion of sophisticated man-made scaffolds approved for TE applications is still to come. There are other factors that justify this expectation. The number of publications found in the pubmed database up to March 2010 was five times higher for “bone TE” as compared to “skin TE”, while the relation between the TE products on the market was reversed (Table 1). At the same time, the peak of patents in bone TE in the 1985–2010 was noted in 2002 while the growing trend for publications in this field persists [6]. This would confirm the patent-publication-market approval order.

**Table 1:** TE products currently available on the medical market

Intended use	Product name (Company)	Cell type used	Scaffold/material used
Wound healing, burns, diabetic and venous leg ulcers	Dermagraft^**®**^ (Shire regenerative medicine, st Helier, Jersey)	Allogenic fibroblasts	Bioabsorbable polyglactin mesh
	Apligraf (Organogenesis Inc., Canton, MA, USA)	Allogenic fibroblasts and keratinocytes	Type I bovine collagen matrix
	MySkin (Altrika Ltd, Sheffield, UK)	Autologous keratinocytes	Silicone coated with a chemically controlled plasma polymer film
	OrCell (Forticell Bioscience, Englewood Cliffs, NJ, USA)	Allogenic fibroblasts and layer of keratinocytes	Type I bovine collagen sponge
	PolyActive (HC Implants BV, leiden, Netherlands)	Autologous cultured fibroblasts and keratinocytes	A compound of polyethyleneoxide terephthalate and polybutylene terephthalate
Cartilage defects	Hyalograft 3D (Fidia Farmaceutici s.p.a.)	Autologous chondrocytes	Hyaluronic acid
	Bioseed-C (BioTissue Technologies, GmbH, Freiburg, Germany)	Autologous chondrocytes	A polyglycolic/polylactic acid and polydioxane based material
	CaReS^®^ (Arthro-Kinetics, Germany)	Autologous chondrocytes	Rat collagen type I
	J-tec, Japan Tissue Engineering Co	Autologous chondrocytes	Atelocollagen gel
	Novocart Inject Novocart 3D (Melsungen, Germany)	Autologous chondrocytes	Polymerizable hydrogel Collagen type I
Bony voids, gaps	FormaGraft (NuVasive, San Diego, CA, USA)	Autologous bone marrow aspirate^a^	Hydroxyapatite, beta-tricalcium phosphate and bovine collagen granules
	Healos^®^ (DePuy Synthes, Warsaw, IN, USA)	Autologous bone marrow aspirate	Type I bovine collagen fibers coated with hydroxyapatite
	Vitoss^®^ Foam (Orthovita/Stryker, Malvern, PA, USA)	Autologous bone marrow aspirate^a^	Β-TCP, Collagen, bioactive glass
	Grafton (Biohorizons, Birmingham, AL, USA	Autologous bone marrow aspirate^a^	DBM
	CopiOs (Zimmer, Warsaw, IN, USA)	Autologous bone marrow aspirate	Autologous bone marrow aspirate

^a^As an option

Another important issue is a growing interest in understanding cell-material interactions [7]. Thus, in a short time we may have the benefit of not only cell tolerating materials, but potentially also cell-instructive materials, which opens new possibilities for advanced ex vivo creation of replacement tissues.

## Cells as the main players

Cell-based concepts for regenerative medicine became possible as a result of availability of cells—successfully manipulated in vitro—with the capability to make tissue. The choice of cell source and cell type(s) is critical to success. In brief, initially, lineage-committed cells harvested from the tissues were considered the major cell type for TE applications. Chondrocytes were among the first cells pursued in the field of TE [8]. Green demonstrated successful rabbit knee cartilage regeneration by chondrocytes seeded on demineralized bone. Soon after, Burke et al. [9] generated the first tissue engineered skin using a collagen matrix and dermal fibroblasts, followed by successful generation of epidermal sheets for large burn wounds [10, 11]. Despite huge progress in cell culture and discovery of alternative, multi- or pluripotent cell types, tissue specific, pre-differentiated (lineage-committed) cells enjoy the attention of the TE field and, for now, are indispensable as a component of commercially available TE products (Table 1). Besides fibroblasts, keratinocytes and chondrocytes, other lineage-committed cells are under investigation and will soon to be employed in regeneration or repair of diverse organ*/*tissue types, including liver [12–14], bladder [15], kidney [16] and pancreas [17]. However, due to low proliferative capacity and accessibility, and insufficient number, primary cells (lineage committed), in some cases, are not a viable choice. Such limitation can be by-passed by use of multipotent stem cells, which can give rise to mesenchymal and non-mesenchymal tissues in vitro and in vivo. Since the discovery of bone marrow mesenchymal stem cells (BMSC) in early 1960s [18] and their subsequent characterization [19–21], there has been continual enthusiasm about the possibilities offered by this type of cells. However, derivation of BMSC requires an invasive procedure. Moreover, with age, differentiation and proliferative potential of BMSCs decline [22, 23]. Therefore, alternative sources from which to isolate multipotent stem cells have been subject to intensive investigation. Among the alternative mesenchymal stem cell sources the most promising are muscles [24], blood [25], placenta [26], amnion [27], umbilical cord blood [28], umbilical cord [29] and adipose tissue [30, 31]. The last seems to be a superior source of stem cells, with greater yield and availability than bone marrow [32]. The unique properties of mesenchymal stem cells, especially their immunosuppressive effect, led to rapid implementation of stem cell clinical trials for a broad spectrum of conditions [33, 34]. Even more is expected from pluripotent stem cells, which have a higher level of plasticity and therefore, a greater potential for diverse applications. Another cell type, embryonic stem cells—ESC [35, 36] discovered in the early 1980s, are controversial because their derivation has been associated with destruction of an early embryo.

Fortunately, the generation of induced pluripotent stem cells (iPS) [37] from somatic cells of human origin has brought forth another potential source of stem cells for TE, cellular therapies and, what seems to be at our fingertips, drug development. The efficiency of obtaining iPS cells is not satisfactory as yet and much effort is still required to use iPS in practice [38]. Still, stem cells obtained by methods free from ethical objections can now be seen on the horizon [39, 40].

Returning to the challenge of engineering a human arm, we propose using autologous, multipotent, so-called adult stem cells, which may be obtained via a minimally invasive procedure, for example, from adipose tissue. At the same time, we look toward use of multipotent iPS in the future. In order to obtain a functional tissue or organ, the cells should undergo differentiation, so that cells of various phenotypes will meet in a single complex system. There are well-documented reports that show that a co-culture is both feasible and effective.

In particular, vascularization of graft might be enhanced by a combination of endothelial cells with osteogenic [41–43] or mesenchymal stem/stromal cells [44–47]. Additionally, endothelial cells may support osteoblast proliferation [48]. Successful osteoblast-osteoclast cell co-culture enabling beneficial cross-talk between those two cell types were also reported [49, 50]. Coexistence of both cell types is necessary for balanced bone remodeling in order to ensure structural integrity. Closely related with osteoclasts are monocytes/macrophages. Their effect on osteogenesis and application in TE is currently being investigated in co-culture with bone marrow stem/stromal cells [51]. Successful limb regeneration may also include reconstruction of osteochondral structures. It has been demonstrated that osteoblasts and chondrocytes influence each other’s biology leading to improved, complex and clinically relevant bone tissue replacement [52–54].

Obviously, the common culture system for the whole arm will be extremely complex. Regardless of the demanding shape and size of the whole bio-device, a separate issue is that the cells of various phenotypes must be put into the common system. Clearly, culture medium preferences, which may be significantly different for the different types of cells needed to regrow a human arm, would not all be addressed under such conditions. Here, we would look to the physicochemical characteristics of the scaffold materials to provide the appropriate cues for regenerative support.

## Significant/substantial role of mechanical factors

There is convincing evidence of a significant effect of substrate stiffness on cell fate. Cell reaction to the rigidity of a support in culture was documented in 1997, when cell spreading, migration and cytoskeletal organization of fibroblasts on polyacrylamide gels of various stiffness was found to vary [55]. Intriguing data showing differentiation of MSCs isolated from bone marrow to three different phenotypes, i.e., neural, osteogenic and myogenic, depending only on the stiffness of the culture substrate without any biochemical stimulation, were published in Cell in 2006 [56]. This finding opens new possibilities for complex constructions such as a whole limb. Progenitor cells seeded on particular parts of the TE construct, having different mechanical characteristics, might receive different signals appropriate for the desired differentiation without exposure to regulatory molecules. This is of crucial importance when the cells of various final/endpoint phenotypes are cultured in a common culture system, i.e., in the same biochemical environment. In addition to the physicochemical characteristics of the biomaterials scaffolds supporting the cells, mechanical inputs will be centrally important for developing the different tissue types.

Mechanical training of tissues being cultured in vitro offers another even more promising possibility. Examples of very well organized ligament structure obtained in a culture of bone marrow cells exposed to a cyclic, well controlled mechanical stimulation were shown by the Kaplan group [57]. Tenogenesis of MSC in response to mechanical stimulation was confirmed also by others [58, 59]. At the same time cyclic mechanical stimulation promotes proliferation and function of smooth muscle cells distributed in 3D scaffolds [60, 61], it may also promote organization of blood vessels in a complex multi-tissue structure.

Promotion of osteoblasts differentiation in response to cyclic mechanical loading applied in vitro has been reported in the Dynacell^®^ culture system with the substrate subjected to deformation [62, 63] and being in contact with implantable materials [64]. Also, osteoblast differentiation was recently reported in 3D systems in which cells were cultured on scaffolds in bioreactors [65–67]; these studies were performed on various scaffolds, including partially demineralized bone matrix [67], fibrin matrix [66] or poly(l-lactic acid) porous scaffolds [65]. They were also performed under different mechanical conditions. Mechanically promoted osteogenic stimulation was achieved in all those situations regardless of the experimental system details. Such findings justify including mechanical stimulation in bone regeneration strategies.

In addition to formation of tendons, ligaments and bone-like tissue in vitro, under applied mechanical forces, tissue engineered cartilage has been obtained under complex mechanical loading [68, 69]. In the case of cartilage, combined compression-shear stimulation was found to be more effective than simple loading [69]. Obtaining cartilage under such conditions was documented not only in culture of animal chondrocytes [68] but also in human MSC culture [69].

Mechanical training, which seems beneficial for maturation and organization of single tissues or tissue-like structures, will be critical for the whole organ. Convincing data showing the significance of cells exercising in vitro for the functionality of the engineered ex vivo bladder are widely known. Obviously, creating the whole human arm in vitro is much more complicated, but not impossible. The brief review given in this paper shows that the necessary tools are already available. We also have access to a wide array of cytocompatible biomaterials representing a broad spectrum of mechanical characteristics. They can be used for the construction of the particular tissues and structures of the human arm and at the same time may serve as temporary scaffolds for cells. Multipotent autologous cells may be obtained via minimally invasive surgery, e.g., from adipose tissue. Their phenotype and function may then be regulated by both physicochemical characteristics of the scaffold and exposure to mechanical training. We have shown here that cells not only tolerate but also respond in a controlled manner to the dynamic conditions of culture in various types of bioreactors. The cells also tolerate co-culture systems, which make possible the construction of organs consisting of cells of various phenotypes. As a result the required tissues may be obtained in vitro due to the responses of the patient’s own cells under the conditions applied to the system.

The mechanical system that simulates the activity and range of motion of the human arm is not necessarily very difficult to obtain. In one of the experiments from the Kaplan group, cells were exposed to mechanical stimulation within an Instron Material Tester [67]. Much more complicated systems dedicated to testing orthopaedic endoprosthesis and conducting orthopaedic kinematics studies under several degrees of freedom are commercially available. They enable using a combination of various types of loading and motions that simulate physiological activity. Such devices for fatigue tests provide a possibility of prolonged observation in a wet environment and might be a perfect basis for constructing a bioreactor for human arm manufacturing in vitro.

## To sum up

The ability to create a full and functional autogenic human limb in an ex vivo bioreactor seems a futuristic dream today. However, reports of the very promising possibilities of engineering tissues in various experimental systems justify our expectation that the goal of a tissue-engineered arm may be reached. Smart, well-characterized biomaterial scaffolds which may influence cells’ fate in a controlled manner, and endogenous extracellular matrix produced by a co-cultured mix of a patient’s own cells and organized under mechanical control in a bioreactor, are suitable tools to make the sophisticated puzzle pieces of an autologous human arm engineered in vitro. We have these tools partly at hand and partly on the near horizon. Perfectly orchestrated collaboration of top class scientists in an interdisciplinary team is indispensable for a successful outcome. In this paper, we strongly emphasize the added value coming from mechanical activation of engineered tissues, which still seems underestimated. Obviously, transferring the promising results from small culture systems to the scale of the whole limb, including the particular difficulty in making interfaces with nerves or blood vessels, will be extremely demanding. Preparing the engineered tissues for anastomose with the appropriate parts of the host tissues via sophisticated surgery would be also challenging. However, examples of spectacular complex surgical procedures, like face transplantation are encouraging in this respect [70]. On the other hand, preparation for the first face transplant by Siemionow took about 20 years. Definitely, we should be very careful in determining the time horizon for producing a live human limb in vitro. We believe that a prototype construction might be realistically achievable within some 20 years. The way from a prototype to the GMP clinically approved technology has to be even longer. Improving the bench-to-bedside pathway is a well-recognized challenge [71, 72]. Therefore, we may expect that new solutions in this area will be elaborated in parallel to the experimental work on regrowing a human arm in vitro. Altogether, we think that hard scientific data and technological possibilities already offered by TE or seen on the horizon justify planning ambitious goals like regeneration of the human arm. Such challenges in turn stimulate further progress in regenerative medicine.

## References

[1] Mason C, Dunnill P (2008). A brief definition of regenerative medicine. Regen Med.

[2] McCarthy M (1996). Bio-engineered tissues move towards the clinic. Lancet.

[3] Jaklenec A, Stamp A, Deweerd E, Sherwin A, Langer R (2012). Progress in the tissue engineering and stem cell industry “are we there yet?”. Tissue Eng Part B Rev.

[4] Regulation (EC) No 1394/2007 of the European Parliament and the Council on advanced therapy medicinal products and amending Directive 2001/83/EC and Regulation (EC) No 726/2004. 2007.

[5] Szpalski C, Wetterau M, Barr J, Warren SM (2012). Bone tissue engineering: current strategies and techniques–part I: scaffolds. Tissue Eng Part B Rev.

[6] Lewandowska-Szumiel M, Wojtowicz J (2011). Bone tissue engineering—a field for new medicinal products?. Curr Pharm Biotechnol.

[7] Unadkat HV, Hulsman M, Cornelissen K, Papenburg BJ, Truckenmuller RK, Carpenter AE (2011). An algorithm-based topographical biomaterials library to instruct cell fate. Proc Natl Acad Sci USA.

[8] Green WT (1977). Articular cartilage repair. Behavior of rabbit chondrocytes during tissue culture and subsequent allografting. Clin Orthop Relat Res.

[9] Burke JF, Yannas IV, Quinby WC, Bondoc CC, Jung WK (1981). Successful use of a physiologically acceptable artificial skin in the treatment of extensive burn injury. Ann Surg.

[10] Gallico GG, O’Connor NE, Compton CC, Kehinde O, Green H (1984). Permanent coverage of large burn wounds with autologous cultured human epithelium. N Engl J Med.

[11] Rheinwald JG, Green H (1975). Serial cultivation of strains of human epidermal keratinocytes: the formation of keratinizing colonies from single cells. Cell.

[12] van de Kerkhove MP, Di Florio E, Scuderi V, Mancini A, Belli A, Bracco A (2002). Phase I clinical trial with the AMC-bioartificial liver. Int J Artif Organs.

[13] Streetz KL (2008). Bio-artificial liver devices–tentative, but promising progress. J Hepatol.

[14] Selden C, Hodgson H (2004). Cellular therapies for liver replacement. Transpl Immunol.

[15] Atala A, Bauer SB, Soker S, Yoo JJ, Retik AB (2006). Tissue-engineered autologous bladders for patients needing cystoplasty. Lancet.

[16] Roy S, Goldman K, Marchant R, Zydney A, Brown D, Fleischman A (2011). Implanted renal replacement for end-stage renal disease. Panminerva Med.

[17] Opara EC, Mirmalek-Sani SH, Khanna O, Moya ML, Brey EM (2010). Design of a bioartificial pancreas(+). J Investig Med.

[18] Becker AJ, Mc CE, Till JE (1963). Cytological demonstration of the clonal nature of spleen colonies derived from transplanted mouse marrow cells. Nature.

[19] Friedenstein AJ, Chailakhjan RK, Lalykina KS (1970). The development of fibroblast colonies in monolayer cultures of guinea-pig bone marrow and spleen cells. Cell Tissue Kinet.

[20] Conget PA, Minguell JJ (1999). Phenotypical and functional properties of human bone marrow mesenchymal progenitor cells. J Cell Physiol.

[21] Pittenger MF, Mackay AM, Beck SC, Jaiswal RK, Douglas R, Mosca JD (1999). Multilineage potential of adult human mesenchymal stem cells. Science.

[22] Nishida S, Endo N, Yamagiwa H, Tanizawa T, Takahashi HE (1999). Number of osteoprogenitor cells in human bone marrow markedly decreases after skeletal maturation. J Bone Miner Metab.

[23] Mueller SM, Glowacki J (2001). Age-related decline in the osteogenic potential of human bone marrow cells cultured in three-dimensional collagen sponges. J Cell Biochem.

[24] Bosch P, Musgrave DS, Lee JY, Cummins J, Shuler T, Ghivizzani TC (2000). Osteoprogenitor cells within skeletal muscle. J Orthop Res.

[25] Zvaifler NJ, Marinova-Mutafchieva L, Adams G, Edwards CJ, Moss J, Burger JA (2000). Mesenchymal precursor cells in the blood of normal individuals. Arthritis Res.

[26] Fukuchi Y, Nakajima H, Sugiyama D, Hirose I, Kitamura T, Tsuji K (2004). Human placenta-derived cells have mesenchymal stem/progenitor cell potential. Stem Cells.

[27] De Coppi P, Bartsch G, Siddiqui MM, Xu T, Santos CC, Perin L (2007). Isolation of amniotic stem cell lines with potential for therapy. Nat Biotechnol.

[28] Lee OK, Kuo TK, Chen WM, Lee KD, Hsieh SL, Chen TH (2004). Isolation of multipotent mesenchymal stem cells from umbilical cord blood. Blood.

[29] Wang HS, Hung SC, Peng ST, Huang CC, Wei HM, Guo YJ (2004). Mesenchymal stem cells in the Wharton’s jelly of the human umbilical cord. Stem Cells.

[30] Zuk PA, Zhu M, Mizuno H, Huang J, Futrell JW, Katz AJ (2001). Multilineage cells from human adipose tissue: implications for cell-based therapies. Tissue Eng.

[31] Zuk PA, Zhu M, Ashjian P, De Ugarte DA, Huang JI, Mizuno H (2002). Human adipose tissue is a source of multipotent stem cells. Mol Biol Cell.

[32] Mizuno H, Tobita M, Uysal AC (2012). Concise review: adipose-derived stem cells as a novel tool for future regenerative medicine. Stem Cells.

[33] Trounson A (2009). New perspectives in human stem cell therapeutic research. BMC Med.

[34] Trounson A, Thakar RG, Lomax G, Gibbons D (2011). Clinical trials for stem cell therapies. BMC Med.

[35] Martin GR (1981). Isolation of a pluripotent cell line from early mouse embryos cultured in medium conditioned by teratocarcinoma stem cells. Proc Natl Acad Sci USA.

[36] Thomson JA, Itskovitz-Eldor J, Shapiro SS, Waknitz MA, Swiergiel JJ, Marshall VS (1998). Embryonic stem cell lines derived from human blastocysts. Science.

[37] Takahashi K, Yamanaka S (2006). Induction of pluripotent stem cells from mouse embryonic and adult fibroblast cultures by defined factors. Cell.

[38] Fu X, Xu Y (2012). Challenges to the clinical application of pluripotent stem cells: towards genomic and functional stability. Genome Med.

[39] Ye L, Swingen C (2012). Zhang J.

[40] Drews K, Jozefczuk J, Prigione A, Adjaye J (2012). Human induced pluripotent stem cells–from mechanisms to clinical applications. J Mol Med (Berl).

[41] Unger RE, Ghanaati S, Orth C, Sartoris A, Barbeck M, Halstenberg S (2010). The rapid anastomosis between prevascularized networks on silk fibroin scaffolds generated in vitro with cocultures of human microvascular endothelial and osteoblast cells and the host vasculature. Biomaterials.

[42] Hofmann A, Ritz U, Verrier S, Eglin D, Alini M, Fuchs S (2008). The effect of human osteoblasts on proliferation and neo-vessel formation of human umbilical vein endothelial cells in a long-term 3D co-culture on polyurethane scaffolds. Biomaterials.

[43] Rouwkema J, de Boer J, Van Blitterswijk CA (2006). Endothelial cells assemble into a 3-dimensional prevascular network in a bone tissue engineering construct. Tissue Eng.

[44] Correia C, Grayson WL, Park M, Hutton D, Zhou B, Guo XE (2011). In vitro model of vascularized bone: synergizing vascular development and osteogenesis. PLoS One.

[45] Kaigler D, Krebsbach PH, West ER, Horger K, Huang YC, Mooney DJ (2005). Endothelial cell modulation of bone marrow stromal cell osteogenic potential. FASEB J.

[46] Grellier M, Granja PL, Fricain JC, Bidarra SJ, Renard M, Bareille R (2009). The effect of the co-immobilization of human osteoprogenitors and endothelial cells within alginate microspheres on mineralization in a bone defect. Biomaterials.

[47] Usami K, Mizuno H, Okada K, Narita Y, Aoki M, Kondo T (2009). Composite implantation of mesenchymal stem cells with endothelial progenitor cells enhances tissue-engineered bone formation. J Biomed Mater Res A.

[48] Leszczynska J, Zyzynska-Granica B, Koziak K, Ruminski S, Lewandowska-Szumiel M (2012). Contribution of endothelial cells to human bone-derived cells expansion in coculture. Tissue Eng Part A.

[49] Tortelli F, Pujic N, Liu Y, Laroche N, Vico L, Cancedda R (2009). Osteoblast and osteoclast differentiation in an in vitro three-dimensional model of bone. Tissue Eng Part A.

[50] Jones GL, Motta A, Marshall MJ, El Haj AJ, Cartmell SH (2009). Osteoblast: osteoclast co-cultures on silk fibroin, chitosan and PLLA films. Biomaterials.

[51] Pirraco RP, Reis RL, Marques AP (2012). Effect of monocytes/macrophages on the early osteogenic differentiation of hBMSCs. J Tissue Eng Regen Med.

[52] Jiang J, Nicoll SB, Lu HH (2005). Co-culture of osteoblasts and chondrocytes modulates cellular differentiation in vitro. Biochem Biophys Res Commun.

[53] Nakaoka R, Hsiong SX, Mooney DJ (2006). Regulation of chondrocyte differentiation level via co-culture with osteoblasts. Tissue Eng.

[54] Chen K, Teh TK, Ravi S, Toh SL, Goh JC (2012). Osteochondral interface generation by rabbit bone marrow stromal cells and osteoblasts coculture. Tissue Eng Part A.

[55] Pelham RJ, Wang Y (1997). Cell locomotion and focal adhesions are regulated by substrate flexibility. Proc Natl Acad Sci USA.

[56] Engler AJ, Sen S, Sweeney HL, Discher DE (2006). Matrix elasticity directs stem cell lineage specification. Cell.

[57] Altman GH, Horan RL, Martin I, Farhadi J, Stark PR, Volloch V (2002). Cell differentiation by mechanical stress. FASEB J.

[58] Kuo CK, Tuan RS (2008). Mechanoactive tenogenic differentiation of human mesenchymal stem cells. Tissue Eng Part A.

[59] Garvin J, Qi J, Maloney M, Banes AJ (2003). Novel system for engineering bioartificial tendons and application of mechanical load. Tissue Eng.

[60] Kim BS, Nikolovski J, Bonadio J, Mooney DJ (1999). Cyclic mechanical strain regulates the development of engineered smooth muscle tissue. Nat Biotechnol.

[61] Sharifpoor S, Simmons CA, Labow RS, Paul Santerre J (2011). Functional characterization of human coronary artery smooth muscle cells under cyclic mechanical strain in a degradable polyurethane scaffold. Biomaterials.

[62] Stanford CM, Morcuende JA, Brand RA (1995). Proliferative and phenotypic responses of bone-like cells to mechanical deformation. J Orthop Res.

[63] Kaspar D, Seidl W, Neidlinger-Wilke C, Ignatius A, Claes L (2000). Dynamic cell stretching increases human osteoblast proliferation and CICP synthesis but decreases osteocalcin synthesis and alkaline phosphatase activity. J Biomech.

[64] Lewandowska-Szumiel M, Sikorski K, Szummer A, Lewandowski Z, Marczynski W (2007). Osteoblast response to the elastic strain of metallic support. J Biomech.

[65] Baas E, Kuiper JH, Yang Y, Wood MA, El Haj AJ (2010). In vitro bone growth responds to local mechanical strain in three-dimensional polymer scaffolds. J Biomech.

[66] Matziolis D, Tuischer J, Matziolis G, Kasper G, Duda G, Perka C (2011). Osteogenic predifferentiation of human bone marrow-derived stem cells by short-term mechanical stimulation. Open Orthop J.

[67] Mauney JR, Sjostorm S, Blumberg J, Horan R, O’Leary JP, Vunjak-Novakovic G (2004). Mechanical stimulation promotes osteogenic differentiation of human bone marrow stromal cells on 3-D partially demineralized bone scaffolds in vitro. Calcif Tissue Int.

[68] Waldman SD, Couto DC, Grynpas MD, Pilliar RM, Kandel RA (2007). Multi-axial mechanical stimulation of tissue engineered cartilage: review. Eur Cell Mater.

[69] Schatti O, Grad S, Goldhahn J, Salzmann G, Li Z, Alini M (2011). A combination of shear and dynamic compression leads to mechanically induced chondrogenesis of human mesenchymal stem cells. Eur Cell Mater.

[70] Siemionow M (2012). Impact of reconstructive transplantation on the future of plastic and reconstructive surgery. Clin Plast Surg.

[71] O’Keefe RJ, Mao J (2011). Bone tissue engineering and regeneration: from discovery to the clinic–an overview. Tissue Eng Part B Rev.

[72] Parenteau N, Hardin-Young J, Shannon W, Cantini P, Russell A (2012). Meeting the need for regenerative therapies I: target-based incidence and its relationship to US spending, productivity, and innovation. Tissue Eng Part B Rev.

